# Preparation of Flame Retardant Polyacrylonitrile Fabric Based on Sol-Gel and Layer-by-Layer Assembly

**DOI:** 10.3390/ma11040483

**Published:** 2018-03-23

**Authors:** Yuanlin Ren, Tongguo Huo, Yiwen Qin, Xiaohui Liu

**Affiliations:** 1 School of Textiles, Tianjin Polytechnic University, No. 399. Binshuixi Road, Xiqing District, Tianjin 300387, China; tongguohuo@163.com (T.H.); yiwenqin@163.com (Y.Q.); 2 Key Laboratory of Advanced Textile Composite, Ministry of Education, Tianjin Polytechnic University, No. 399. Binshuixi Road, Xiqing District, Tianjin 300387, China; 3 School of Materials Science and Engineering, Tianjin Polytechnic University, No. 399. Binshuixi Road, Xiqing District, Tianjin 300387, China; xiaohuilau@163.com

**Keywords:** PAN fabric, sol-gel, layer-by-layer assembly, phytic acid, flame retardancy

## Abstract

This paper aims to develop a novel method, i.e., sol-gel combined with layer-by-layer assembly technology, to impart flame retardancy on polyacrylonitrile (PAN) fabrics. Silica-sol was synthesized via the sol-gel process and acted as cationic solution, and phytic acid (PA) was used as the anionic medium. Flame-retardant-treated PAN fabric (FR-PAN) could achieve excellent flame retardancy with 10 bilayer (10BL) coating through layer-by-layer assembly. The structure of the fabrics was characterized by X-ray photoelectron spectroscopy (XPS) and Fourier transform infrared spectroscopy (FTIR). The thermal stability and flame retardancy were evaluated by thermogravimetric (TG) analysis, cone calorimetry (CC) and limiting oxygen index (LOI). The LOI value of the coated fabric was up to 33.2 vol % and the char residue at 800 °C also increased to 57 wt %. Cone calorimetry tests revealed that, compared to the control fabric, the peak of heat release rate (PHRR) and total heat release (THR) of FR-PAN decreased by 66% and 73%, respectively. These results indicated that sol-gel combined with layer-by-layer assembly technique could impart PAN fabric with satisfactory flame-retardant properties, showing an efficient flame retardant strategy for PAN fabric.

## 1. Introduction

Polyacrylonitrile (PAN) fibers, one of the most important synthetic fibers, occupy a unique position in textile applications thanks to their good warmth retention and profound resistance to light, radiation, mildew, etc. However, PAN fibers have a very low limiting oxygen index (LOI) value (17 vol %) and so belong to a group of highly flammable fibers [1,2,3]. As a result, PAN fibers or fabrics are easy to ignite and burn vigorously and release dense smoke once they are ignited. Thus, some flame-retardant methods have been developed for improving the flame retardancy of acrylonitrile copolymer, PAN fibers or PAN fabrics.

Generally, blending [4,5], copolymerization [6,7], finishing [2] and chemical modification [8,9,10] are the common techniques to obtain flame retardant PAN fibers or fabrics. Quite recently, nanotechnological approaches have attempted to impart flame retardancy to different fabrics comprising a variety of fibre types, such as layer-by-layer assembly technique, sol-gel process, and plasma deposition [11], etc.

Layer-by-layer (LbL) assembly is a kind of simple, potentially environmentally friendly and versatile approach, which can impart flame retardancy to the surface of different kinds of substrates (e.g., fabrics, thin films and foam), involving the alternative deposition of positively and negatively charged species [12,13]. Various flame-retardant nano-coatings have been successfully fabricated by this method based on the electrostatic attractions between alternate layers. Using this technique, some research groups have started to develop a new intumescent system based on LbL assembly coatings in order to obtain flame-retardant fabrics. For example, an intumescent fire-retardant coating consisting of cationic chitosan and anionic ammonium polyphosphate (APP) was developed by Fang [14,15] and was successfully deposited on cotton fabric by LbL assembly. The increased char residue at 700 °C in air and decreased peak of heat release rate (PHRR) of coated cotton fabrics was evidence of excellent flame-retardant efficiency. They also prepared a polyhexamethylene guanidine phosphate–ammonium polyphosphate (PHMGP-APP) bilayer nanocoating in order to enhance the flame retardancy and antimicrobial properties of cotton fabrics. Alongi [16] deposited a nanostructured LbL coating, including APP and octapropyl ammonium polyhedral oligomericsilsequioxane (POSS), onto acrylic fabrics. The acrylic fabrics were homogeneously covered and the melt dripping phenomenon of fabrics was completely suppressed with 4 or 6 bi-layers coatings.

Another effective nanotechnological approach, the sol-gel technique, is usually based on a two-step reaction (hydrolysis and condensation), starting from semimetal alkoxides to form an organic–inorganic coating. The silicon hydrogel derived from this sol-gel process may be combined with phosphorus and/or nitrogen-containing additives in order to impart flame retardancy to fabrics [17,18,19,20]. In our previous work, an organic–inorganic hybrid silica coating doped with either polyphosphoric acid (PPA) [21] or phytic acid (PA) and urea [22] was prepared by the sol-gel process and coated on polyacrylonitrile (PAN) fabrics to impart flame retardancy. The results showed that the coated PAN fabric achieved satisfactory flame retardancy. In addition, Alongi [23,24,25] reported considerable work to improve the flame retardancy of cotton fabrics based on the pure silica sol or phosphorus-, nitrogen-containing silica sols. Thus, the sol-gel technique had been widely applied for the flame-retardant finishing on different fabrics.

However, to the best of our knowledge, there has been no research on obtaining flame-retardant PAN fabrics through the combination of sol-gel process and layer-by-layer assembly technique. In our previous work [21,22], either PPA or PA was directly added into the sol-gel system and stirred evenly, then the blended substrates were coated onto PAN fabric and dried to obtain flame retardant PAN fabric. In this work, we developed a novel coating based on the combination of a sol-gel process and layer-by-layer assembly technology to improve the flame-retardant performance of PAN fabrics. The cationic silicon hydrogel solution was firstly prepared using 3-aminapropyltriethoxysilane (KH-550) as precursor by the sol-gel process. Then, the anionic solution was prepared from a PA solution, which acted as a bio-based, high phosphorus-containing compound. The PAN fabric was coated with silicon hydrogel and PA alternately, which is different from our previous work, i.e., blending coating, and the structure, fire resistance and thermal stability of the modified PAN fabric were investigated in detail.

## 2. Experimental Section

### 2.1. Materials

Polyacrylonitrile (PAN) fiber with 95 wt % acrylonitrile and 5 wt % vinyl acetate was supplied by Jilin Chemical Fiber Co., Jilin, China. Scoured plain-woven PAN fabrics (400 g/m^2^) were prepared by the School of Textiles, Tianjin Polytechnic University, Tianjin, China. Ethanol and sodium hydroxide were supplied by Tianjin Fengchuan Chemical Reagent Co., Ltd., Tianjin, China. 3-aminapropyltriethoxysilane (KH-550) and phytic acid (PA, 70 wt % aqueous solution) were obtained from Nanjing Xiezun Chemical Reagent Co., Ltd., Nanjing, China.

### 2.2. Preparation of Cationic Solution Via Sol-Gel Process

The cationic solution was prepared by the sol-gel technique using KH-550 as precursor. Firstly, a specific amount of 30 wt % of KH-550 was added into a mixing solution of alcohol and deionized water (1:1, *v*/*v*). Secondly, the pH of the above solution was adjusted to 11 with the addition of NaOH aqueous solution (0.1 M). Finally, the obtained solution was stirred for 4 h at room temperature to prepare the cationic silica-sol solution.

### 2.3. Preparation of Anionic Solution

The anionic solution was prepared using 11 wt % of PA aqueous solution and its pH was adjusted to 7 with NaOH solution (0.1 M).

### 2.4. Preparation of Flame Retardant PAN Fabrics through Layer-by-Layer Assembly

**Scheme 1:**
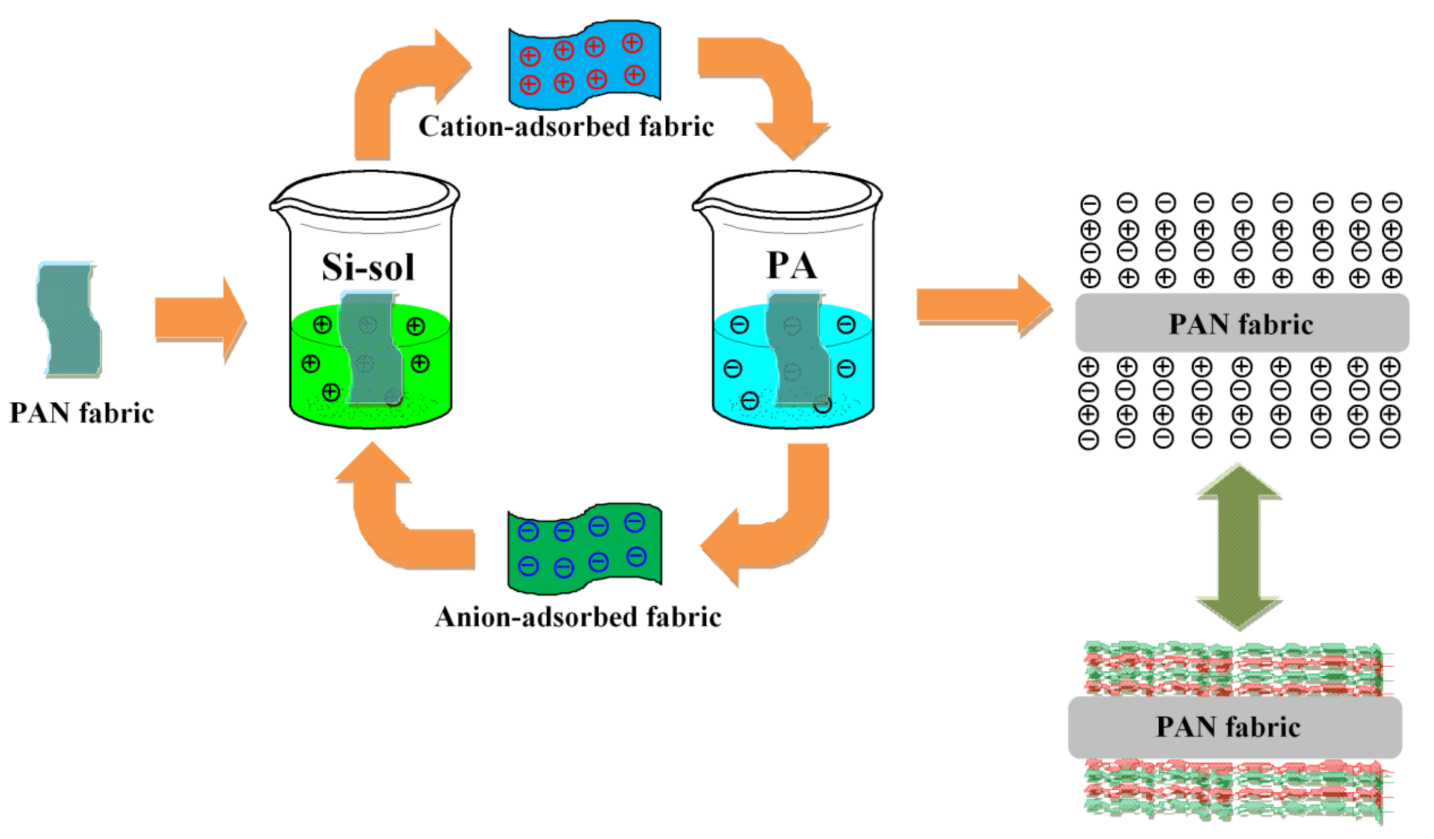
The schematic of the layer-by-layer assembly process. PAN: polyacrylonitrile.

All coated PAN fabrics were prepared with the same process, as shown in Scheme 1, in which a piece of PAN fabric was firstly impregnated with the silica-sol for 5 min, rinsed with deionized water, squeezed to remove excess silica-sol solution and dried in air; subsequently, the cation-adsorbed PAN fabric was dipped into the PA solution for 5 min, then the excess PA solution was squeezed followed by washing with deionized water and drying in air to obtain the anion-adsorbed PAN fabric. In this way, one bilayer (BL) of silica-sol and PA was successfully coated onto the surface of the fibers within the PAN fabric. In order to obtain the desired number of bilayers on PAN fabric, the fabric was alternately immersed into the cationic and anionic solutions. Finally, the coated PAN fabrics were dried in vacuum oven at 60 °C for 30 min followed by curing in oven at 160 °C for 5 min. The flame-retardant PAN fabric (FR-PAN) was obtained when 10 BLs of coating was deposited.

### 2.5. Characterizations

The Fourier transform infrared spectra (FTIR) of fabrics were measured by a Vector22 spectrometer (Bruker, Billerica, MA, USA) with the resolution ratio of 4 cm^−1^. The wavenumber ranged from 400 cm^−1^ to 4000 cm^−1^ with 120 scans.

Solid-state ^29^Si NMR spectroscopy of the silicon gel was recorded through a Bruker AV400 NMR spectrometer (400 MHz) (Bruker Biospin GmbH, Billerica, MA, USA) using 160 scans with a pulse width of 5 μs and a recycle delay of 10 s. Tetramethylsilane (TMS) was used as a reference for chemical shifts.

The surface elemental composition of fabrics and the residual char of coated fabrics after burning were tested using an X-ray photoelectron spectrometer (ThermoFisher co., Waltham, MA, USA).

Thermo-gravimetric analysis (TGA) of fabrics (at. 5.7 mg) was performed on a STA-409PC TGA thermo-analyzer (Netzsch, Germany) from 20 °C to 800 °C at a heating rate of 10 °C/min under air atmosphere (air flux: 50 mL/min).

The surface morphology of the fabrics before and after coating as well as the chars were observed by scanning electron microscopy (FE-SEM; S-4800, Hitachi co., Tokyo, Japan) with the beam voltage of 10 kV. Before testing, they were coated with gold for 2 min (E1045, Hitachi ion sputter, Tokyo, Japan).

The combustion performance of fabrics was evaluated by a cone calorimeter (FTT, East Grinstead, UK) according to ISO 5660-1 under an irradiative heat flux of 35 kW/m^2^ in horizontal configuration. All the specimens with the dimension of 100 mm × 100 mm were placed in aluminum foil to protect the edges and back of the sample and maintained in the correct configuration by a metallic grid welded at intersections [26]. The experiments were repeated five times for each sample examined to ensure reproducible data. All the specimens were conditioned at 23 ± 1 °C for 48 h under the condition of 50% relative humidity in a climatic chamber before combustion tests. Time to ignite (TTI), Heat release rate (HRR) and corresponding peak (PHRR), total heat release (THR), total smoke production (TSP), smoke production rate (SPR) and corresponding peak (pSPR) and fire growth rate index (FIGRA) were all evaluated. FIGRA values are obtained by dividing the PHRR values by the time required to reach the PHRR event (PHRR/tPHRR), providing an estimation of both the spread rate and the size of a fire, and the contribution to fire growth of materials [27,28]. Meanwhile, the residues of samples after testing were photographed by a digital camera (Power-Shot A2000 IS, Canon Inc., Tokyo, Japan).

Limiting oxygen index (LOI) measured by using a HC-2 oxygen index apparatus according to GB 5454-85.

## 3. Results and Discussion

### 3.1. FTIR Analysis

**Figure 1:**
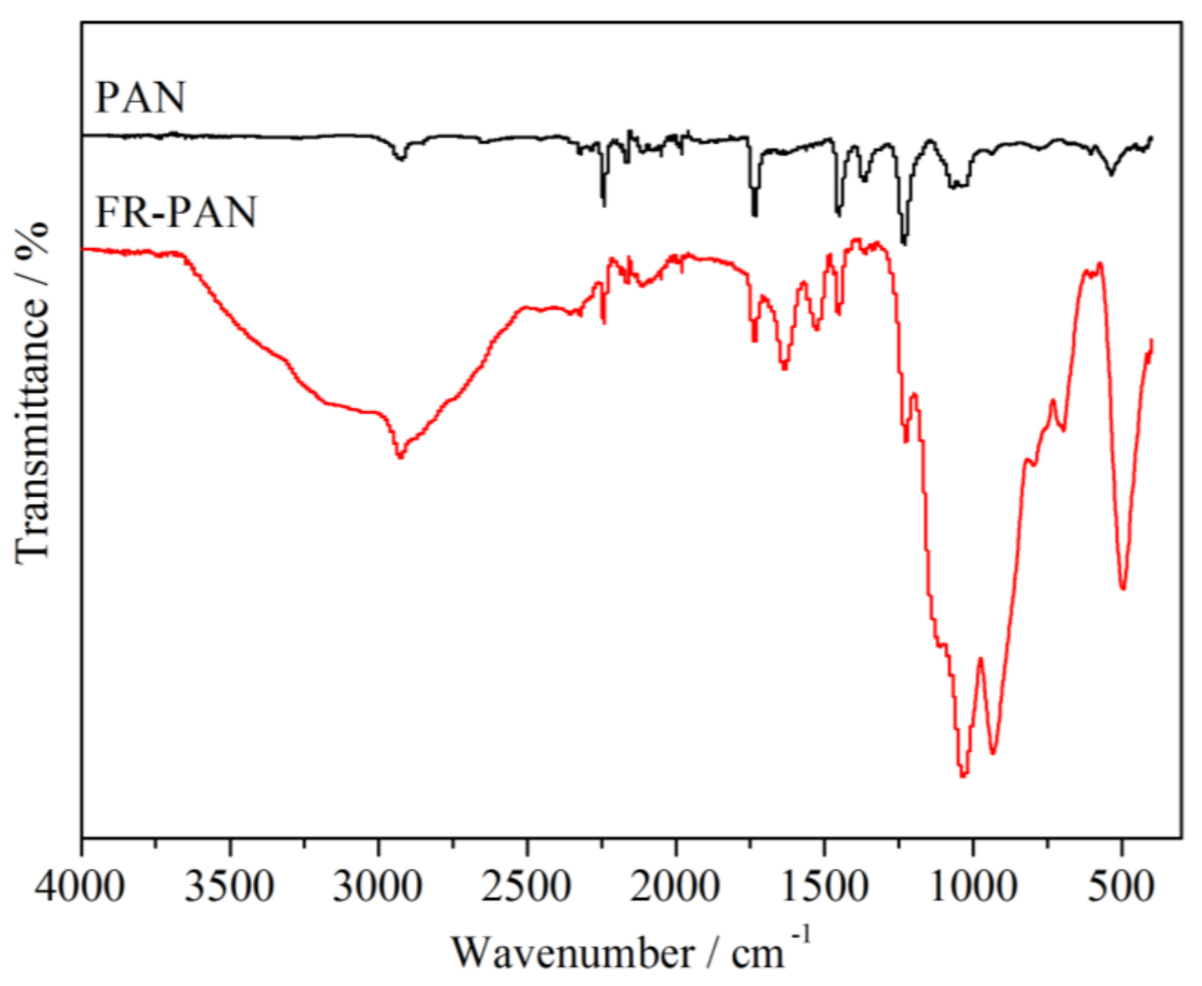
FTIR spectra of PAN and flame-retardant PAN fabrics.

The FTIR spectra of original PAN and flame-retardant PAN (FR-PAN) fabrics are shown in Figure 1.

For the blank fabric, the adsorption peaks of stretching vibrations at 2242 cm^−1^ (C≡N), 1732 cm^−1^ (C=O of ester group), 1446 cm^−1^ (C-H bending in CH_2_) and 1245 cm^−1^ (C-N bending) are clearly observed, which are the characteristic peaks of the copolymerized PAN fibers of acrylonitrile and vinyl acetate. Compared to the control fabric, the coated PAN fabric showed new adsorption bands at 1030 cm^−1^ and 694 cm^−1^, corresponding to the asymmetrical and symmetrical stretching vibration of Si-O-Si respectively [13,18,21,22,29]. Furthermore, another two new bands at 1230 cm^−1^ and 796 cm^−1^ are observed in the spectrum of FR-PAN, which are attributed to P=O and P-O stretching vibration, respectively [21,30,31,32]. The results demonstrate that the silica-sol and phytic acid (PA) have been successfully coated on the surface of PAN fabric by using layer-by-layer assembly technology.

### 3.2. ^29^Si NMR Analysis

**Figure 2:**
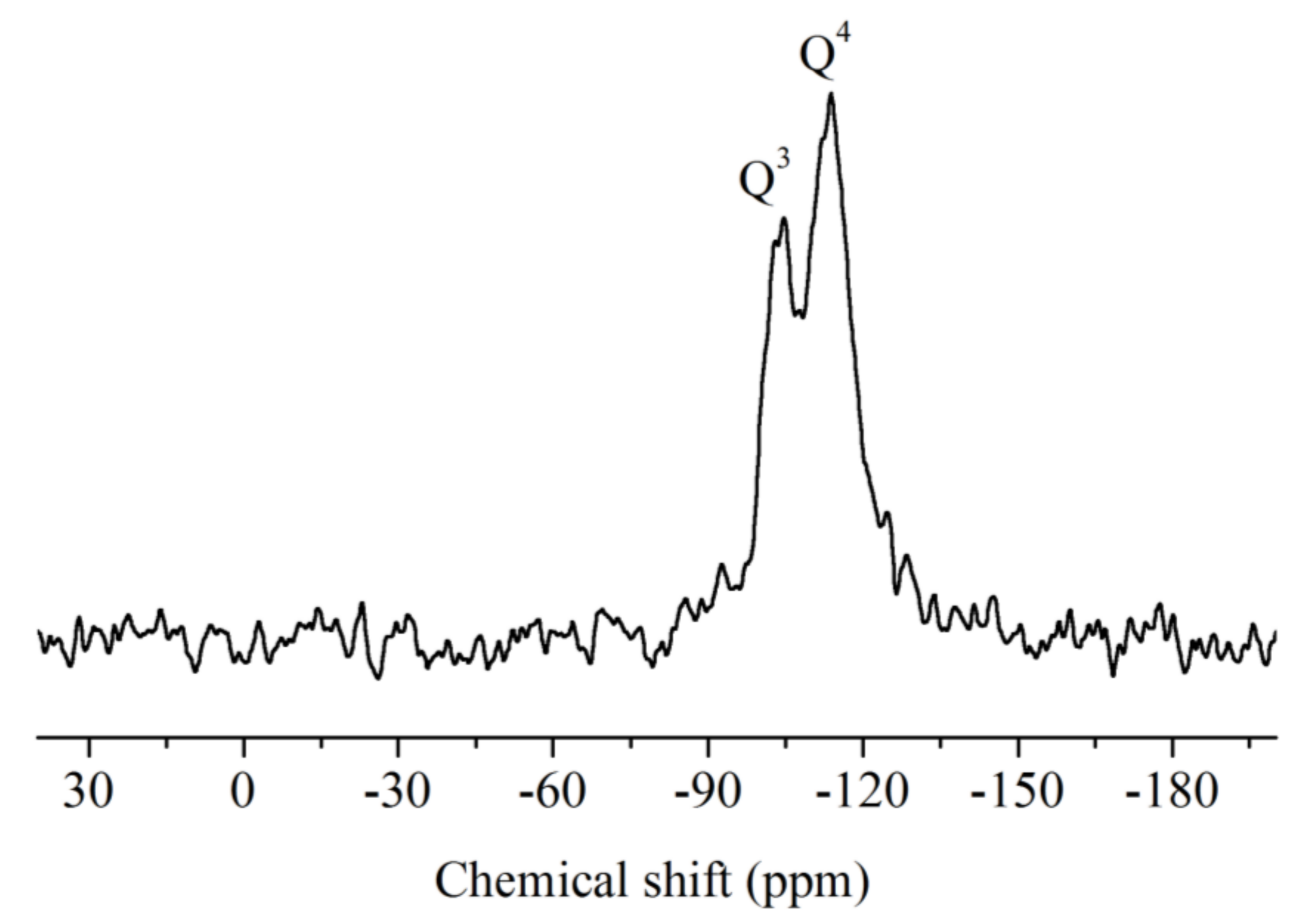
Solid-state ^29^Si NMR spectrum of the silicon coating.

Figure 2 displays the solid-state ^29^Si NMR spectrum of the silicon coating. The silicate units are usually represented by Q^n^, referring to a silicon with n bridging oxygens to other silicon atoms [33,34]. For the solid-state ^29^Si NMR spectrum, the signals are located between −100 and −120 ppm, which indicates the presence of a ^29^Si nucleus in a tetrahedral oxygen environment. The silicon species of Q^3^ and Q^4^ are −104 and −113 ppm, respectively, which originate from the hydrolysis and condensation of 3-aminapropyltriethoxysilane (KH-550). As can be seen in the figure the fraction of Q^4^ peak centered at −113 ppm is relatively strong, which indicates relative complete condensation of inorganic network [35,36]. And the condensation degree of the hybrid sol reached 95%, indicating high cross-linking extent of KH-550.

### 3.3. XPS Analysis

**Figure 3:**
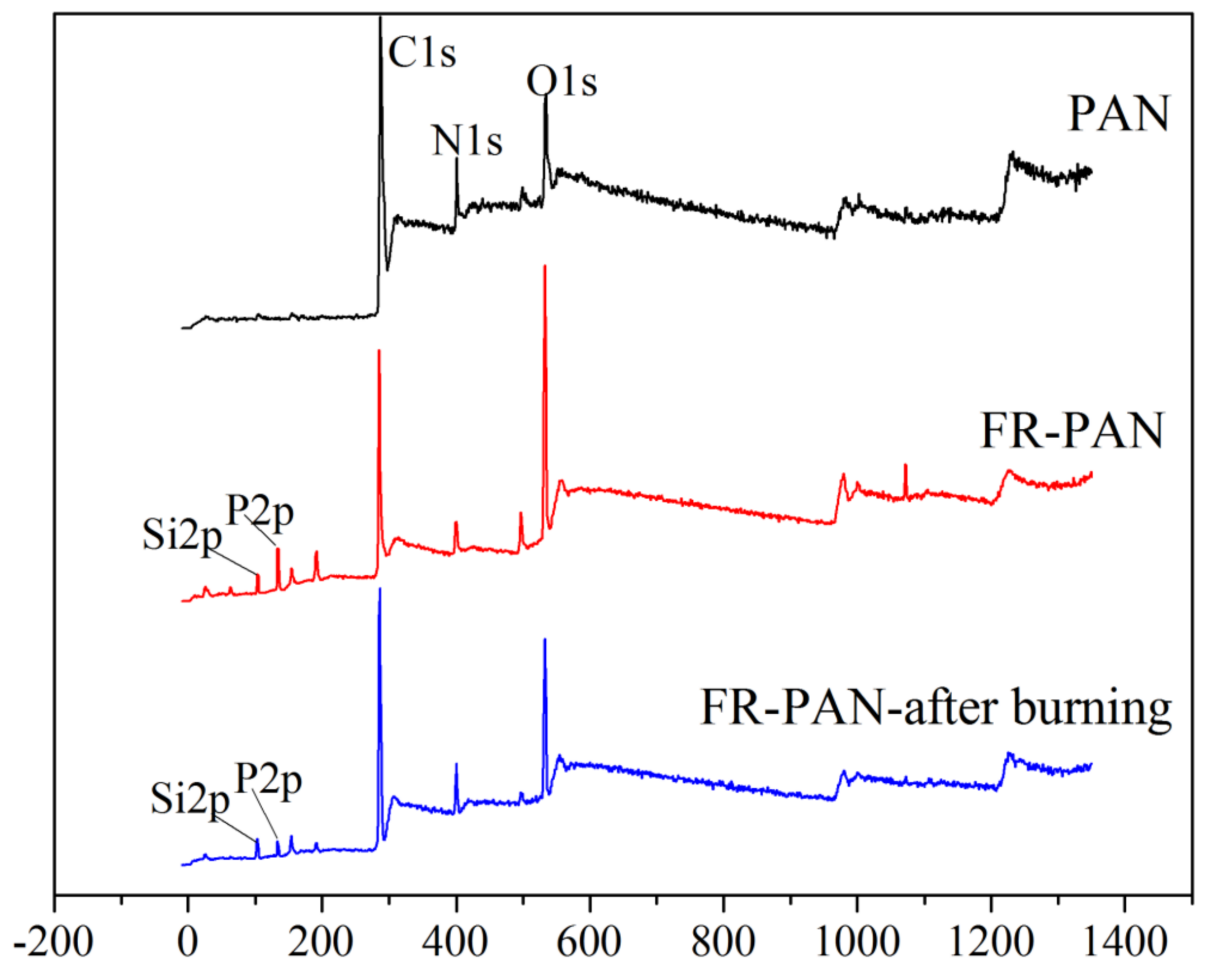
XPS spectra of PAN fabric, FR-PAN fabric before and after burning.

Figure 3 displays the XPS spectra of PAN, FR-PAN and FR-PAN after LOI test. The content of corresponding elements is listed in Table 1. As shown in Figure 3, for the spectra of all the samples, the peaks appear around 281, 403 and 529 eV corresponding to C1s, N1s and O1s, respectively. But for the spectrum of FR-PAN, peaks arise at 100 and 151 eV attributed to Si2p and P2p, respectively. Furthermore, the content of Si as well as P elements reaches to 7.99 wt % and 5.36 wt %, respectively (as listed in Table 1), indicating that the bilayer coating containing PA and silica-sol has been successfully deposited onto the surface of PAN fabric. After burning, the content of Si in FR-PAN has remained relatively stable (5.80 wt %), furthermore, the content of C has increased from 54.21 wt % to 67.12 wt %, which may be due to the fact that the formation of SiO_2_ inorganic fire-retardant barrier and char layer resulting from the catalytic effect of Si, P and N protects the fabric from decomposition effectively, meanwhile, during burning/heating, hydrogen will be lost including as NH_3_ as will some oxygen as CO_2_, which could explain part of the increase in carbon: as a result, the content of C increases greatly.

### 3.4. SEM Analysis

**Figure 4:**
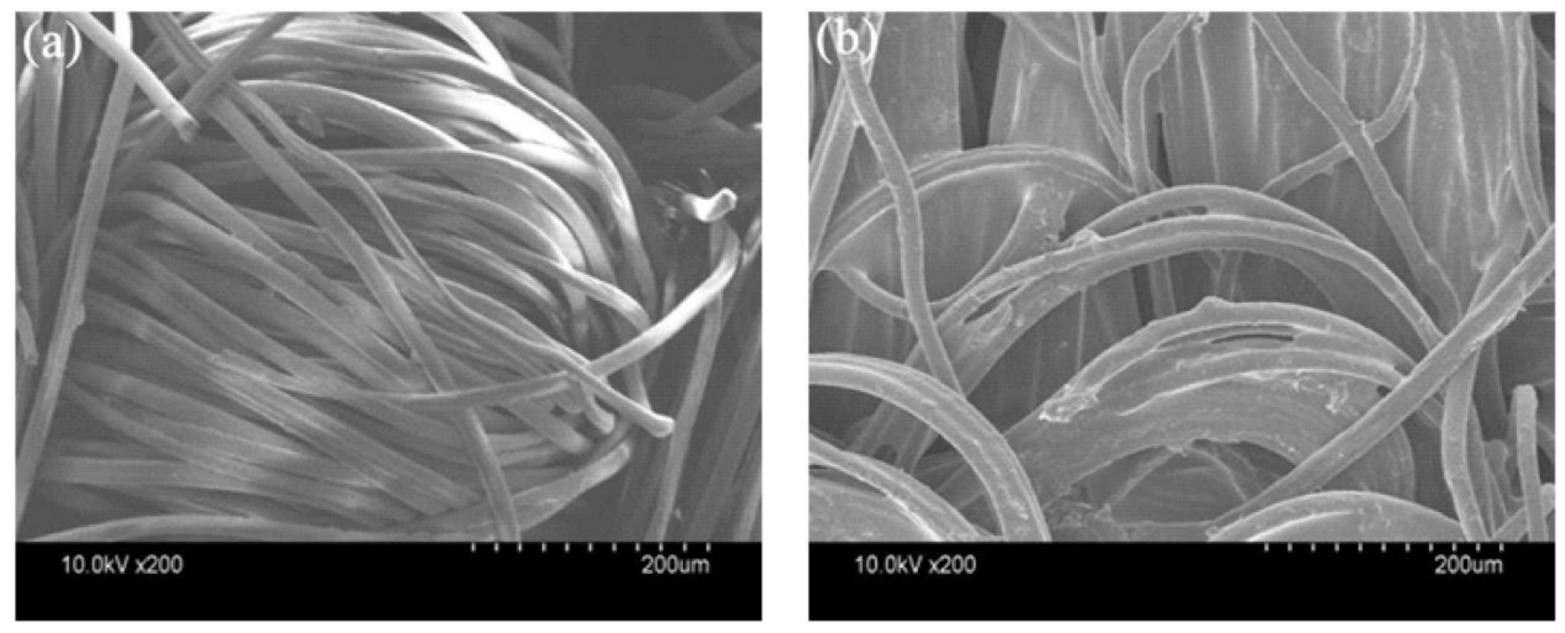
SEM photos of PAN and flame-retardant PAN fabrics. (**a**) PAN fabric; (**b**) flame retardant fabric.

SEM is used to observe the surface morphology of untreated and treated PAN fabrics, as shown in Figure 4. The original PAN fibers display smooth surfaces. However, when the surface of the fibers within the fabric is coated with 10BLs of silicon-sol and PA, the fiber surfaces become rougher and a large amount of surface coating appears, with some causing adhesions between fibers. 

### 3.5. Thermal Stability Analysis

**Figure 5:**
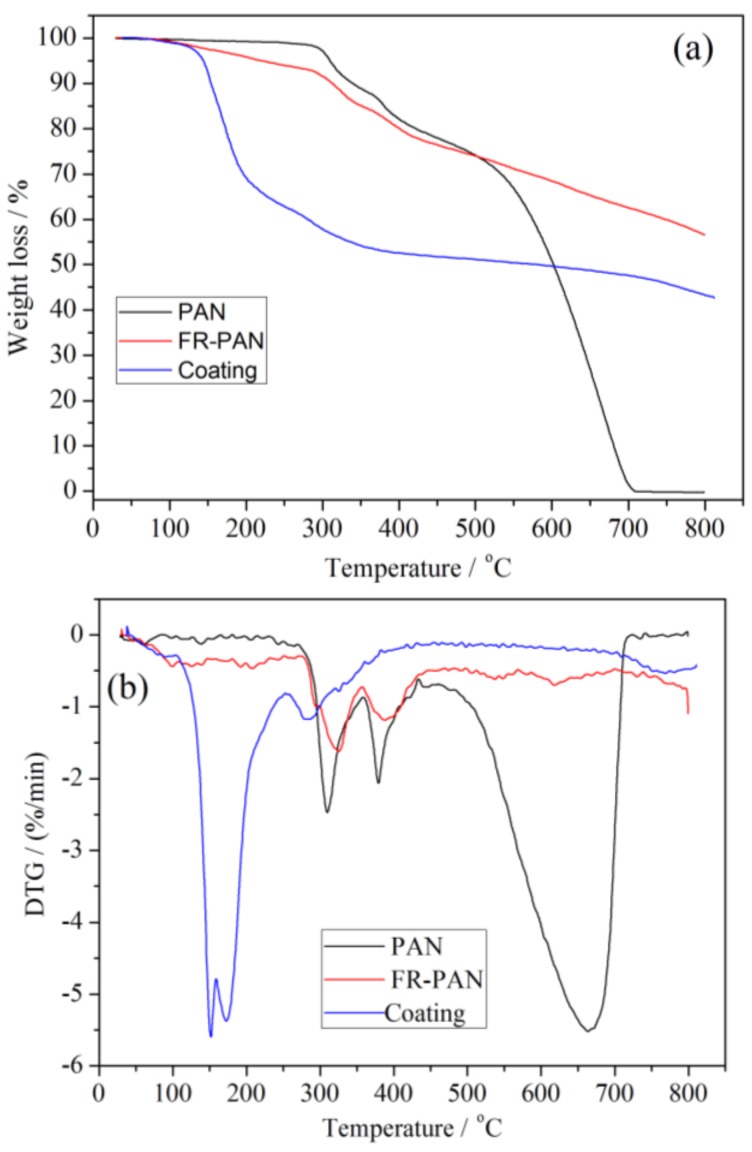
TG-DTG curves of PAN, FR-PAN and coating in air.

**Figure 6:**
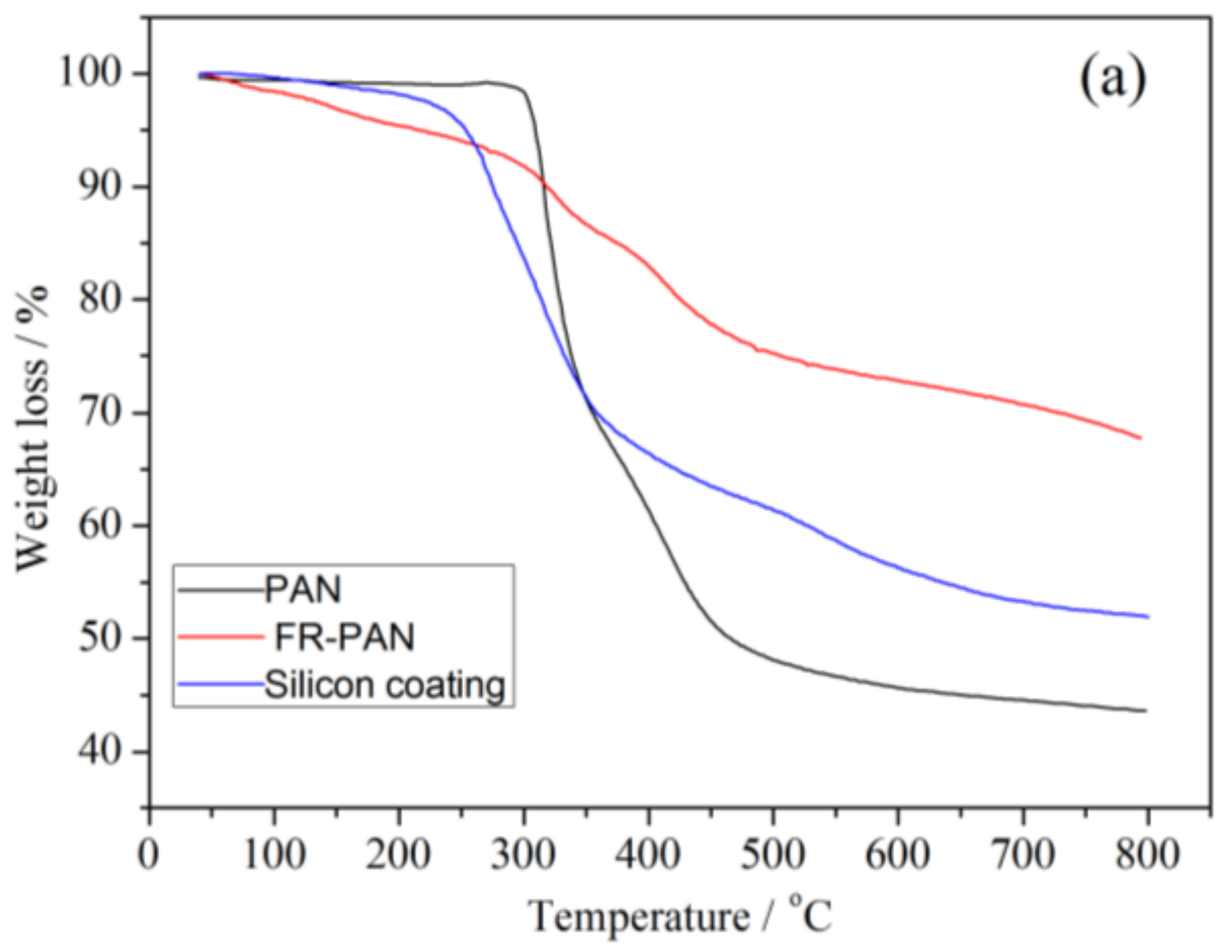

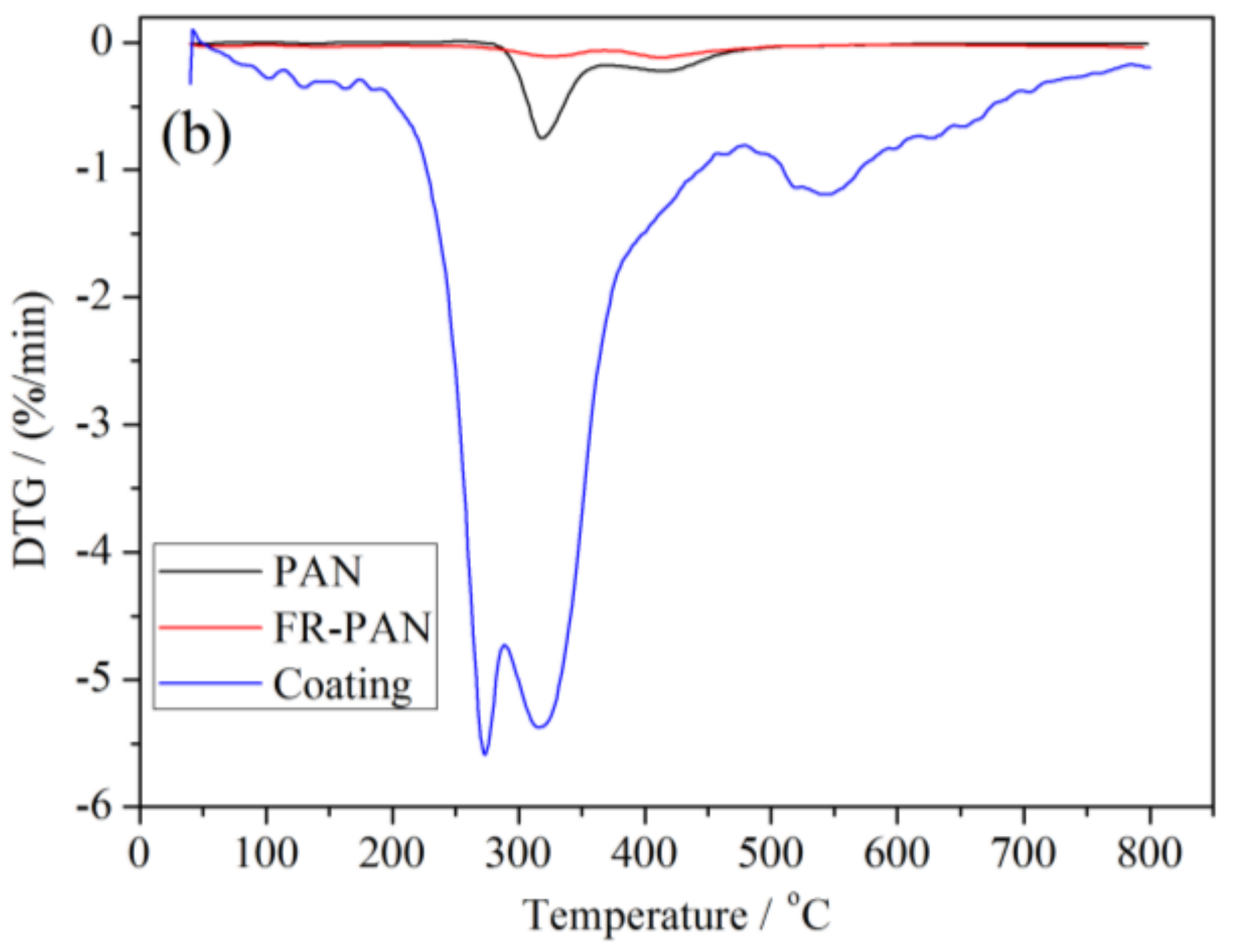
TG-DTG curves of PAN, FR-PAN and coating in N_2._

The TG and derivative thermogravimetric (DTG) curves of PAN, FR-PAN and coating in air and nitrogen are shown in Figure 5 and Figure 6, respectively, and the detailed data are summarized in Table 2. 

As for the curve of original PAN fabric, there are three decomposition stages corresponding to the cyclization, decomposition–carbonization and thermo-oxidation of char [37,38]. The first stage starts from 297 °C to 358 °C following a little weight loss due to the release of HCN and NH_3_ during the cyclization reaction. The second stage ranged from 358 °C to 443 °C and was defined to be a decomposition-carbonization process of cyclic structure with the volatilization of H_2_. And the last stage corresponds to the thermo-oxidation (485–720 °C) of the char residue produced in the first two stages which would be further decomposed and oxidized, and no char residue left at 800 °C. 

For the decomposition of the coating, the TG plot shows three mass loss steps. The first event occurred between 120 °C and 159 °C with a mass loss of about 13 wt % and corresponded to the removal of the combined water of phytic acid and silicon gel. The second one, with a mass loss of about 21 wt %, is attributed to the carbonization process and dehydration due to the decomposition of OH groups present in the phytic acid and the dehydration of silicon gel. The last one is due to the process of thermal decomposition of phytate groups and the elimination of elemental carbon fromed in the previous steps, and the mass loss is 13 wt %. The residue at 800 °C is around 43 wt %, attributed to the generated SiO_2_.

Similarly, the main decomposition process of FR-PAN also has three decomposition stages ranging from 280 °C to 356 °C, 358 °C to 448 °C and 500 °C to 700 °C, respectively. It can be clearly seen that the onset temperature of cyclization has decreased, indicating that the introduction of PA can catalyze the cyclization of PAN macromolecules resulting in the formation of char layer [21,39,40]. Moreover, the char layer can effectively hinder the exchange of heat between the fabric and environment. The decomposition–carbonization process is also similar to that of the control sample. However, the weight loss in the third stage is remarkably less compared with that of the original PAN fabric, and the residue at 800 °C reaches to 57 wt %, illustrating that the coating containing Si-sol and PA is able to inhibit the decomposition process of PAN. In addition, on the one hand, the SiO_2_ generated from silicon-containing compounds during the combustion process acts as a good inorganic flame retardant barrier to prevent the residue from further oxidative decomposition and increase the stability of the residue decomposed from PAN substrate, on the other hand, the generated SiO_2_ will remain in the residue, in other words, it contributes to the increase in the residue. Hence, the fabric deposited with 10BLs obtains an excellent flame retardancy. 

The decomposition of the samples in nitrogen is different from that in air. As shown in Figure 6, for the origin PAN fabric, there are two main decomposition steps ranging from 273 °C to 360 °C and 366 °C to 550 °C with a weight loss of about 32 wt % and 24 wt %, respectively, and 44 wt % residue was left. The two weight losses are attributed to cyclization dehydrogenation and decomposition–carbonization.

FR-PAN undergoes a similar decomposition process to that in air. The first weight loss ranges from 296 °C to 380 °C and its maximum weight loss rate occurs at 330 °C, which is a little higher than that in air (324 °C). The second one starts from 384 °C to 483 °C with the maximum weight loss rate being at 405 °C. On the one hand, the Si-O-Si bonds enhance the thermal stability of PAN; On the other hand, the generated SiO_2_ network together with the resultant char layer of PAN fabric promoted by PA form a compact barrier to isolate heat and oxygen and protect FR-PAN from decomposing. As a result, the residue is about 68 wt %, which is much higher than that of control PAN (57 wt %).

As far as the coating is concerned, the TG plot also shows three mass loss steps. The peaks of the three weight losses are 270 °C, 330 °C and 550 °C, respectively, which are higher than those in air, showing higher thermal stability. The residue at 800 °C is around 52 wt %.

### 3.6. Flame Retardancy

The cone calorimeter (CC) was used to evaluate the combustion behavior of the original PAN and FR-PAN fabrics.

**Figure 7:**
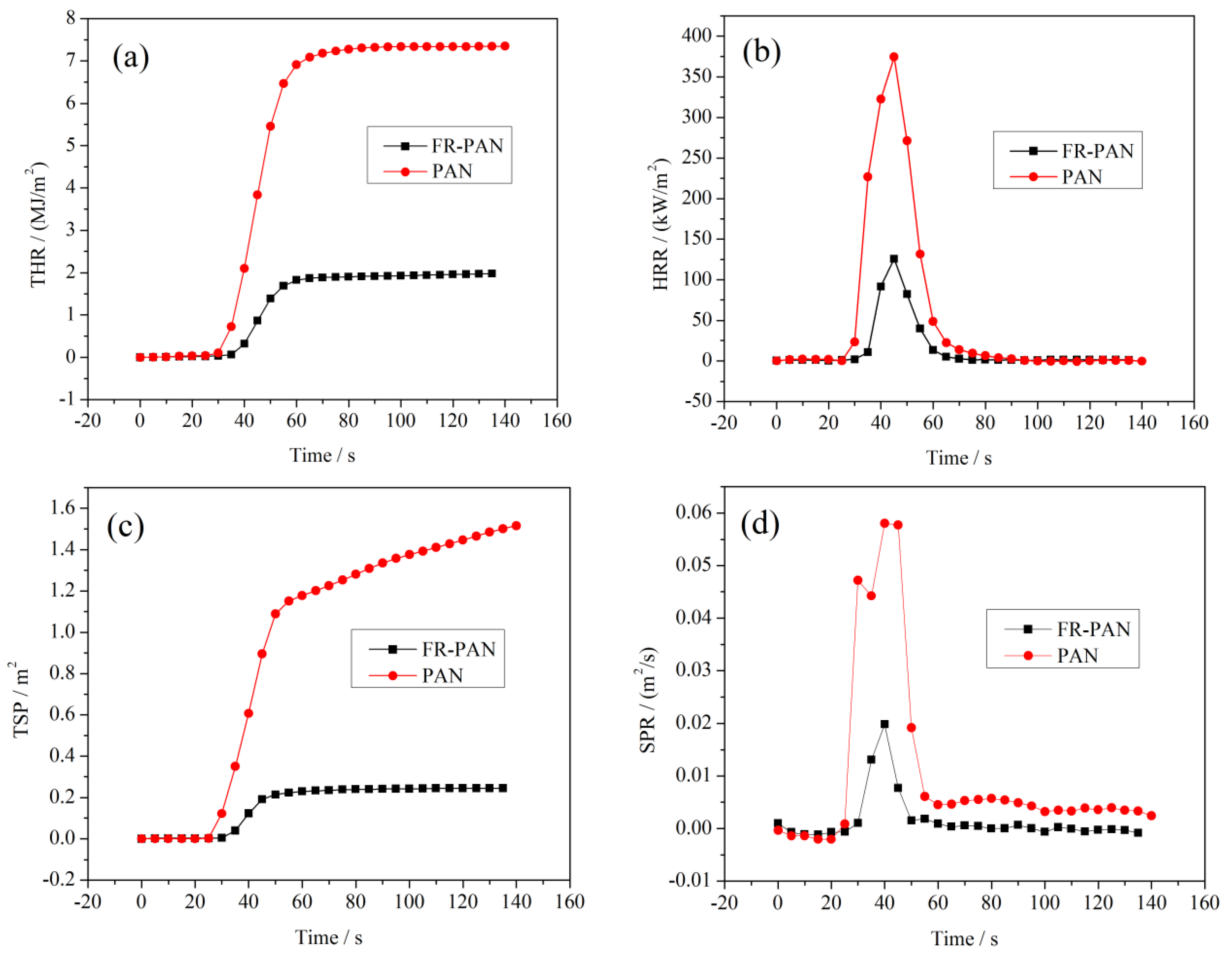
The total heat release (THR) (**a**); heat release rate (HRR) (**b**); total smoke production (TSP) (**c**) and smoke production rate (SPR) (**d**) curves of PAN and flame-retardant PAN fabrics.

The time to ignition (TTI), total heat release (THR), heat release rate (HRR), total smoke production (TSP) and smoke production rate (SPR) are shown in Figure 7. The collected data are listed in Table 3. Compared to the original PAN, the peak of heat release rate (PHRR) and THR of FR-PAN decreases from 374 kW/m^2^ to 126 kW/m^2^ with a reduction of 66% and from 7.3 MJ/m^2^ to 2.0 MJ/m^2^ with 73% reduction, respectively, which demonstrates that the HRR of the fabric with 10BLs coating will be inhibited because of the deposition of PA and silica-sol. As shown in Table 3, the average mass loss rate (aMLR) of FR-PAN (0.01 g/s) shows a significant decrease, however, the char residue increases a lot, which is consistent with the result of TG test. Furthermore, the fire growth rate index (FIGRA) of FR-PAN drops from 8.3 (kW/m^2^)/s to 2.8 (kW/m^2^)/s with a reduction about 66%. The greatly decreased FIGRA value illustrates that the coating effectively inhibits the spread of fire as well as the further burning of PAN fabric [21].

As for the TSP and SPR curves of PAN and FR-PAN (Figure 7c,d), compared with the control sample, the peak of SPR (PSPR) of FR-PAN decreases from 0.06 m^2^/s to 0.02 m^2^/s with a 67% reduction, and the TSP value also decreases from 1.5 m^2^ to 0.2 m^2^ with 87% reduction. This may be due to the fact the coating containing PA and silica-sol can effectively suppress the smoke production. It is interesting to find that except the time to PHRR, all the other properties of the PAN fabric treated with sol-gel combined with layer-by-layer assembly technique are better than those of the PAN fabric modified with sol-gel method by using polyphosphoric acid (PPA) as the blending fire retardant, as shown in Table 3.

**Figure 8:**
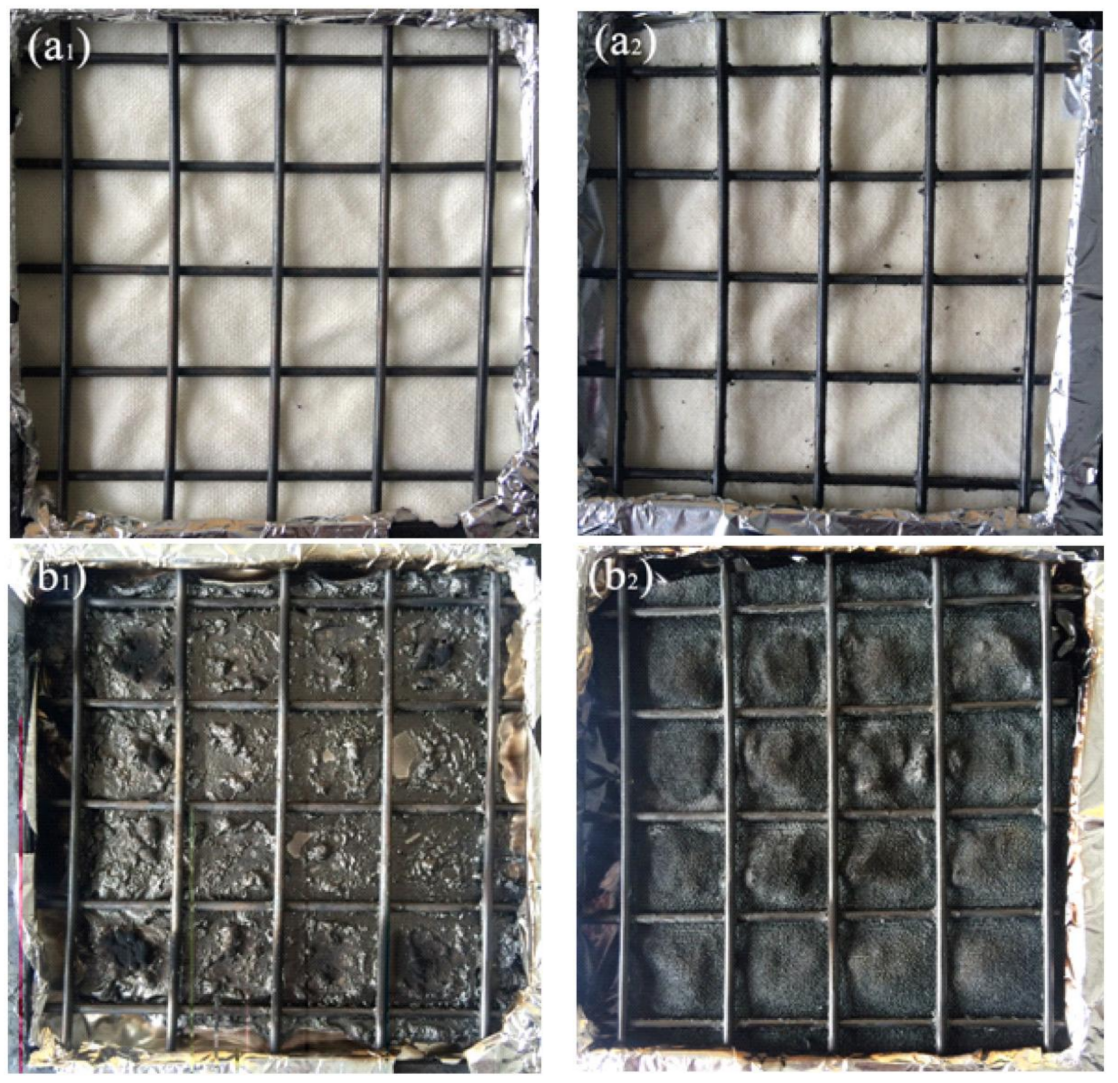
The digital photographs of the fabrics before and after CC testing. (**a_1_**) PAN fabric; (**a_2_**) FR-PAN fabric; (**b_1_**) PAN fabric after burning; (**b_2_**) FR-PAN fabric after burning.

The digital photographs of different fabrics before and after CC testing are displayed in Figure 8. From Figure 8b_1_, it can be clearly seen that the PAN fabric after CC testing forms a thin, fragile and incomplete char layer accompanying a greatly decrease of the char residue. However, one continuous and dense char layer is obtained for FR-PAN after CC testing (Figure 8b_2_). As a result, the char layer can act as an insulator to hinder the heat transfer of oxygen and fabric, which also even can prevent the further burning of PAN fabrics.

**Figure 9:**
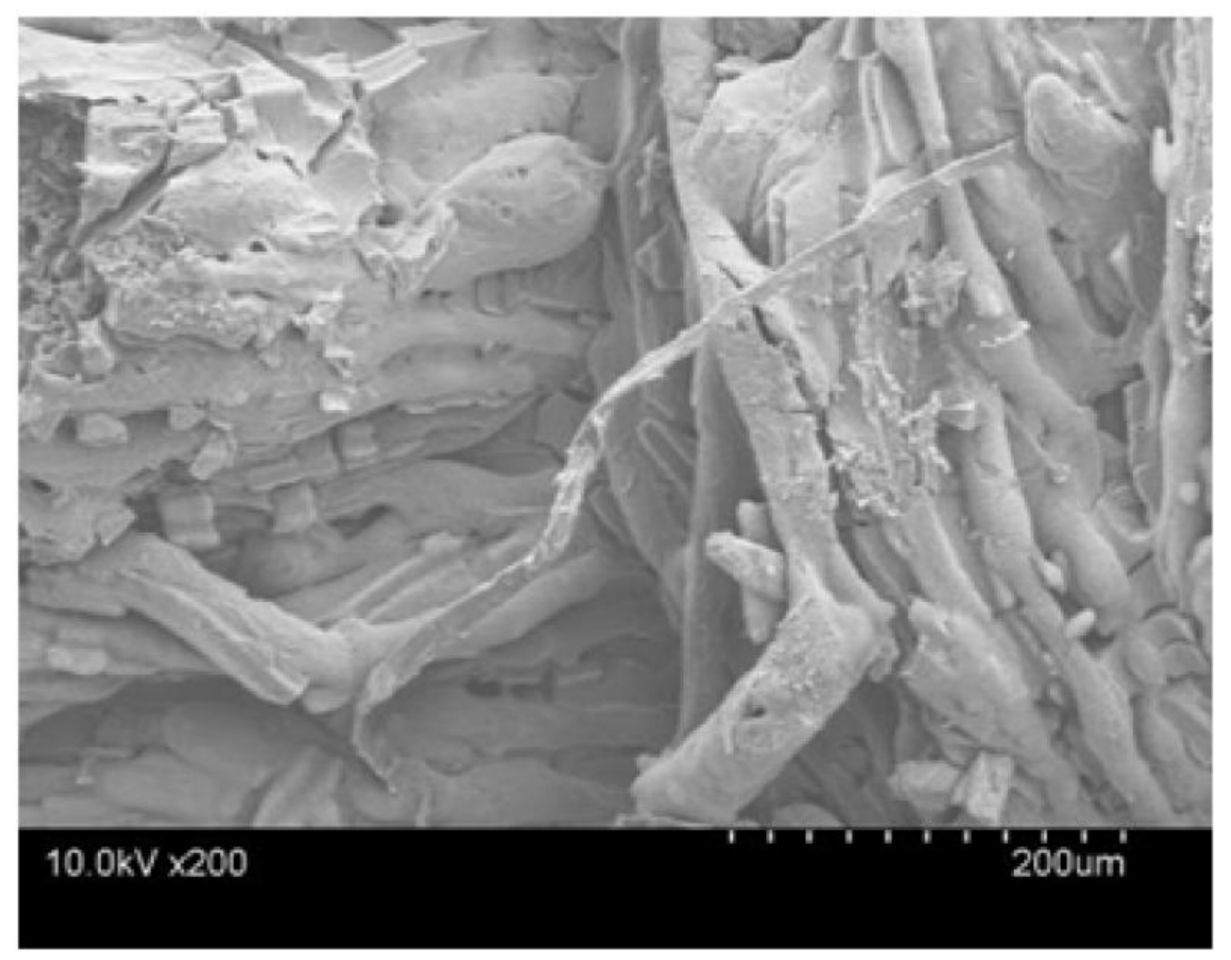
SEM photos of FR-PAN after CC test.

Figure 9 shows the SEM picture of FR-PAN after CC testing. As shown in Figure 9, the structure of the fabric and the morphology of fibers keep a complete structure after burning because of the presence of coating on its surface. Obviously, the fibers within the fabric become thick accompanying lots of cracks. This indicates that the addition of PA can catalyze the cyclization process of PAN, and lead to the formation of char-layer. Moreover, the synthetic effect occurred between Si and P is also able to effectively improve the residue of FR-PAN.

As for the LOI value of PAN and FR-PAN fabric, the FR-PAN possesses a higher LOI value (33.2 vol %) compared to the untreated PAN sample (17 vol %), demonstrating that the flame retardancy of PAN has been greatly improved.

## 4. Conclusions

In this paper, a flame-retardant coating containing silica-sol and phytic acid has been successfully deposited onto PAN fabric following the combination of sol-gel process and layer-by-layer technology. The fabric coated with 10BL of silica-PA possessed good flame retardancy, showing a higher LOI value of 33.2 vol %. Furthermore, according to the analysis of TG-DTG and CC, the PHRR and THR of FR-PAN were decreased by 66% and 73% compared to original PAN, respectively; the value of pSRR and TSP also exhibited an apparent decrease. However, the residues of FR-PAN at 800 °C reached up to 57 wt %: this was due to the silica-PA coating that could inhibit the exchange between fabric and oxygen. Consequently, the combination of sol-gel and layer-by-layer assembly technology presented a huge advantage in the aspect of enhancing flame retardant of PAN fabric.

## Figures and Tables

**Table 1:** Element content of different fabrics. FR-PAN: flame-retardant PAN; LOI: limiting oxygen index.

Samples	C/wt %	O/wt %	N/wt %	P/wt %	Si/wt %
PAN	66.53	21.31	9.12	-	-
FR-PAN	54.21	25.77	4.68	7.99	5.36
FR-PAN after LOI test	67.12	16.25	7.70	3.12	5.80

**Table 2:** Thermogravimetric (TG) data of PAN, FR-PAN and coating in air and N_2_ atmosphere.

Samples		T_onset, 5%_ (°C)	T_max1_ (°C)	T_max2_ (°C)	T_max3_ (°C)	Residue (wt %)
PAN	air	297	308	381	664	0
	N_2_	310	320	420	-	44
FR-PAN	air	281	324	392	-	57
	N_2_	201	330	405	-	68
Coating	air	150	154	175	286	43
	N_2_	260	270	330	550	52

**Table 3:** Parameters of PAN and FR-PAN by cone calorimetry test. PHRR: peak of heat release rate; aMLR: average mass loss rate; FIGRA: fire growth rate index.

Samples	TTI (s)	PHRR (kW/m^2^)	Time to PHRR (s)	THR (MJ/m^2^)	PSPR (m^2^/s)	TSP (m^2^)	aMLR (g/s)	FIGRA ((kW/m^2^)/s)	Residue (wt %)
PAN	25	374	45	7.3	0.06	1.5	0.02	8.3	38
FR-PAN	28	126	45	2.0	0.02	0.2	0.01	2.8	51
Si/P-coated fabric [21]	27	168	45	3.1	0.03	0.3	0.02	3.7	47

## References

[1] Xue T.J., Mckinney M.A., Wilkie C.A. (1997). The thermal degradation of polyacrylonitrile. Polym. Degrad. Stab..

[2] Yaman N. (2009). Preparation and flammability properties of hybrid materials containing phosphorous compounds via sol-gel process. Fire Mater..

[3] Xu L., Cheng B.W., Ren Y.L., Lu Y.C. (2010). Research progress of flame retardant polyacrylonitrile and its fiber. J. Tex. Res..

[4] Ren Y.L., Cheng B.W., Xu L., Jiang A.B., Lu Y.C. (2009). Fire-retardant copolymer of acrylonitrile with *O*,*O*-diethyl-*O*-allyl thiophosphate. J. Appl. Polym. Sci..

[5] Ren Y.L., Wang L.J., Liu T.T. (2016). Preparation and performance of halogen-free fire retardant polyacrylonitrile fiber. Polym. Mater. Sci. Eng..

[6] Wyman P., Crook V., Ebdon J., Hunt B., Joseph P. (2006). Flame-retarding effects of dialkyl-p-vinylbenzyl phosphonates in copolymers with acrylonitrile. Polym. Int..

[7] Zhang J., Horrocks A.R., Hall M.E. (2010). Flammability of polyacrylonitrile and its copolymers. IV. The flame retardant mechanism of ammonium polyphosphate. Fire Mater..

[8] Sahiner N., Pekel N., Guven O. (1999). Radiation synthesis, characterization and amidoximation of N-vinyl-2-pyrrolidone/acrylonitrile interpenetrating polymer networks. React. Funct. Polym..

[9] Tsafack M.J., Levalois-Grützmacher J. (2006). Plasma-induced graft-polymerization of flame retardant monomers onto PAN fabrics. Surf. Coat. Technol..

[10] Yan X., Zhou W., Zhao X., Xu J., Liu P. (2015). Preparation, flame retardancy and thermal degradation behaviors of polyacrylonitrile fibers modified with diethylenetriamine and zinc ions. J. Therm. Anal. Calorim..

[11] Alongi J., Carosio F., Malucelli G. (2014). Current emerging techniques to impart flame retardancy to fabrics: An overview. Polym. Degrad. Stab..

[12] Liang S.Y., Neisius N.M., Gaan S. (2013). Recent developments in flame retardant polymeric coatings. Prog. Org. Coat..

[13] Wang X., Romero M.Q., Zhang X.Q., Wang R., Wang D.Y. (2015). Intumescent multilayer hybrid coating for flame retardant cotton fabrics based on layer-by-layer assembly and sol–gel process. RSC Adv..

[14] Fang F., Xiao D., Zhang X., Meng Y., Cheng C., Bao C., Ding X., Cao H., Tian X. (2015). Construction of intumescent flame retardant and antimicrobial coating on cotton fabric via layer-by-layer assembly technology. Surf. Coat. Technol..

[15] Fang F., Zhang X., Meng Y., Gu Z., Bao C., Ding X., Li S., Chen X., Tian X. (2015). I ntumescent flame retardant coatings on cotton fabric of chitosan and ammonium polyphosphate via layer-by-layer assembly. Surf. Coat. Technol..

[16] Carosio F., Alongi J. (2016). Influence of layer by layer coatings containing octapropylammonium polyhedral oligomeric silsesquioxane and ammonium polyphosphate on the thermal stability and flammability of acrylic fabrics. J. Anal. Appl. Pyrolysis.

[17] Brancatelli G., Colleoni C., Massafra M.R., Rosace G. (2011). Effect of hybrid phosphorus-doped silica thin films produced by sol-gel method on the thermal behavior of cotton fabrics. Polym. Degrad. Stab..

[18] Colleoni C., Donelli I., Freddi G., Guido E., Migani V., Rosace G. (2013). A novel sol-gel multi-layer approach for cotton fabric finishing by tetraethoxysilane precursor. Surf. Coat. Technol..

[19] Zhang Q., Zhang W., Huang J., Lai Y., Xing T., Chen G., Jin W., Liu H., Sun B. (2015). Flame retardance and thermal stability of wool fabric treated by boron containing silica sols. Mater. Des..

[20] Aksit A., Onar N., Kutlu B., Sergin E., Yakin I. (2016). Synergistic effect of phosphorus, nitrogen and silicon on flame retardancy properties of cotton fabric treated by sol-gel process. Int. J. Cloth. Sci. Technol..

[21] Ren Y.L., Zhang Y., Zhao J.Y., Wang X.L., Zeng Q., Gu Y.T. (2016). Phosphorus-doped organic–inorganic hybrid silicon coating for improving fire retardancy of polyacrylonitrile fabric. J. Sol-Gel Sci. Technol..

[22] Ren Y.L., Zhang Y., Gu Y.T., Zeng Q. (2017). Flame retardant polyacrylonitrile fabrics prepared by organic-inorganic hybrid silica coating via sol-gel technique. Prog. Org. Coat..

[23] Alongi J., Colleoni C., Malucelli G., Rosace G. (2012). Hybrid phosphorus-doped silica architectures derived from a multistep sol–gel process for improving thermal stability and flame retardancy of cotton fabrics. Polym. Degrad. Stab..

[24] Alongi J., Colleoni C., Rosace G., Malucelli G. (2013). Phosphorus- and nitrogen-doped silica coatings for enhancing the flame retardancy of cotton: Synergisms or additive effects?. Polym. Degrad. Stab..

[25] Alongi J., Colleoni C., Rosace G., Malucelli G. (2014). Sol–gel derived architectures for enhancing cotton flame retardancy: Effect of pure and phosphorus-doped silica phases. Polym. Degrad. Stab..

[26] Nazare S., Kandola B., Horrocks A.R. (2002). Use of cone calorimetry to quantify the burning hazard of apparel fabrics. Fire Mater..

[27] Wang D.Y., Liu X.Q., Wang J.S., Wang Y.Z., Stec A.A., Hull T.R. (2009). Preparation and characterisation of a novel fire retardant PET/α-zirconium phosphate naocomposite. Polym. Degrad. Stab..

[28] Kandare E., Luangtriratana P., Kandola B.K. (2014). Fire reaction properties of flax/epoxy laminates and their balsa-core sandwich composites with or without fire protection. Composites B.

[29] Grancaric A.M., Botteri L., Alongi J., Tarbuk A. (2016). Silica precursor as synergist for cotton flame retardancy. Int. J. Cloth. Sci. Technol..

[30] Ren Y.L., Cheng B.W., Jiang A.B., Lu Y.C., Xu L. (2010). Thermal degradation kinetics of poly(*O*,*O*-diethyl-*O*-allylthiophosphate-co-acrylonitrile) in nitrogen. J. Appl. Polym. Sci..

[31] Penczek S., Pretula J., Kubisa P., Kaluzynski K., Szymanski R. (2015). Reactions of H_3_PO_4_ forming polymers. Apparently simple reactions leading to sophisticated structures and applications. Prog. Polym. Sci..

[32] Wang L.H., Ren Y.L., Wang X.L., Zhao J.Y., Zhang Y., Zeng Q., Gu Y.T. (2016). Fire retardant viscose fiber fabric produced by graft polymerization of phosphorus and nitrogen-containing monomer. Cellulose.

[33] Sinko K., Meiszterics A., Rohonczy J., Kobzi B., Kubuki S. (2017). Effect of phosphorus precursors on the structure of bioactive calcium phosphate silicate systems. Mater. Sci. Eng. C.

[34] Irwin A.D., Holmgren J.S., Jonas J. (1987). Solid-state 29Si NMR study of polycondensation during heat treatment of sol-gel derived silicas. Mater. Lett..

[35] Karatas S., Hosgor Z., Kayaman-Apohan N., Gungor A. (2009). Preparation and characterization of phosphine oxide containing organosilica hybrid coatings by photopolymerization and sol-gel process. Prog. Org. Coat..

[36] Chen X.W., Liang C.X., Guan J.P., Yang X.H., Tang R.C. (2018). Flame retardant and hydrophobic properties of novel sol-gel derived phytic acid/silica hybrid organic-inorganic coatings for silk fabric. Appl. Surf. Sci..

[37] Surianarayanan M., Vijayaraghavan R., Raghavan K.V. (1998). Spectroscopic investigations of polyacrylonitrile thermal degradation. J. Polym. Sci. Part A Polym. Chem..

[38] Martin S.C., Liggat J.J., Snape C.E. (2001). In situ NMR investigation into the thermal degradation and stabilisation of PAN. Polym. Degrad. Stab..

[39] Qin O., Cheng L., Wang H., Li K. (2008). Mechanism and kinetics of the stabilization reactions of itaconic acid-modified polyacrylonitrile. Polym. Degrad. Stab..

[40] Liu Y., Pan Y.T., Wang X., Acuna P., Zhu P., Wagenknecht U., Heinrich G., Zhang X.Q., Wang R., Wang D.Y. (2016). Effect of phosphorus-containing inorganic–organic hybrid coating on the flammability of cotton fabrics: Synthesis, characterization and flammability. Chem. Eng. J..

